# Investigating the Preservative Effect of Edible Coating Based on Chitosan‐Gelatin Containing *Satureja bachtiarica* and *Nepeta binaludensis* Essential Oils Nanoemulsions on the Physicochemical, Microbial and Sensory Properties of Burger During Cold Storage

**DOI:** 10.1002/fsn3.72302

**Published:** 2026-08-28

**Authors:** Mahsa Nakhaei, Homa Baghaei, Fariborz Nahidi, Parnian Pezeshki, Ahmadreza Abedinia

**Affiliations:** ^1^ Department of Food Science and Technology, Da.C. Islamic Azad University Damghan Iran; ^2^ Department of Food Science & Technology (Quality control & Hygien) Varastegan Institute for Medical Sciences Mashhad Iran

**Keywords:** active coating, Bakhtiari savory, Binalodi oregano, meat product, nanoemulsion, shelf life

## Abstract

This study investigated the effectiveness of chitosan‐gelatin (CG) based coatings enriched with free and nanoemulsion forms of *Satureja bachtiarica* (SE) and *Nepeta binaludensis* (NE) essential oils on the quality of beef burgers during 12‐day refrigerated storage. GC–MS analysis identified 37 and 50 phytochemicals in SE and NE, respectively, with carvacrol and 1,8‐cineole being prominent compounds. The nanoemulsions were characterized by a spherical shape, a size range of 65.6–122.4 nm, low, polydispersity index (0.286–0.391), zeta potential between −26.7 and −38.2 mV, and high encapsulation efficiency (83.96%–85.31%). While nano‐encapsulation slightly reduced total phenol content, it enhanced the essential oils' antimicrobial activity. Over the storage period, the pH, total volatile basic nitrogen (TVB‐N), and microbial load of all burger treatments increased, while L* and a* values decreased. Compared with the control, burgers coated with the SE nanoemulsion exhibited a 52.9% reduction in TVB‐N (14.90 vs. 31.62 mg N/100 g) and a 38.8%, 42.7%, 39.7%, and 57.1% reduction in viable mesophilic bacteria, psychrotrophic bacteria, LAB, and coliform counts, respectively, on day 12. Moreover, the NE nanoemulsion reduced mold and yeast counts by 48.2% compared with the control (2.54 vs. 4.90 log CFU/g). The nanoemulsion forms of the essential oils demonstrated superior antimicrobial efficacy throughout storage, with the SE nanoemulsion‐coated burgers exhibiting the highest activity at the end of the trial. Sensory evaluation confirmed that these active coatings maintained burger acceptability. These findings suggest that CG‐based coatings enriched with SE and NE nanoemulsions are a promising solution for extending the shelf life of beef burgers.

## Graphical abstract

A chitosan–gelatin edible coating containing nanoemulsions of *Satureja bachtiarica* and *Nepeta binaludensis* essential oils was developed and applied to burgers during refrigerated storage. The active coating improved microbial stability and helped maintain the physicochemical and sensory quality of burgers, demonstrating its potential for extending refrigerated shelf life.

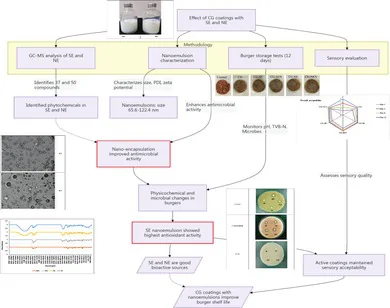

## 1 Introduction

Meat and meat products are highly nutritious but are susceptible to microbial spoilage and lipid oxidation because of their high moisture content and abundant nutrients, resulting in quality deterioration, economic losses, and potential health risks (Gorzin et al. 2024; Hashemi, Kaveh, et al. 2023). Active edible coatings based on renewable biopolymers have emerged as sustainable preservation strategies by providing antimicrobial protection and reducing physicochemical deterioration during storage (Khalid and Arif 2022). Among these biopolymers, chitosan exhibits excellent film‐forming ability and inherent antimicrobial activity, whereas gelatin improves film integrity and barrier performance, making chitosan–gelatin composites attractive carriers for natural preservatives (Abedinia et al. 2018; Cao et al. 2020; Erceg et al. 2023; Lv et al. 2023).

Growing consumer demand for clean‐label foods has accelerated the replacement of synthetic preservatives with plant‐derived bioactive compounds, particularly essential oils (Gorzin et al. 2026; Isvand et al. 2024). *Satureja bachtiarica* and *Nepeta binaludensis* are two Iranian endemic medicinal plants rich in phenolic compounds and terpenoids with well‐documented antioxidant and antimicrobial activities (Fathimoghaddam et al. 2020; Mahalleh et al. 2020, 2021; Pirastehfard et al. 2021). However, the volatility and environmental instability of essential oils limit their direct application in foods (Radi et al. 2023).

Nanoemulsion technology effectively improves the stability, dispersion, and controlled release of essential oils (Abedinia, Serri, et al. 2026; Bakhshalipoor et al. 2026), thereby enhancing their functional performance in edible coatings. Ultrasonication is one of the most effective and widely applied techniques for producing food‐grade nanoemulsions with improved physicochemical stability (Ahmadreza Abedinia, Khajehali Chaleshtori, et al. 2026; Azarashkan et al. 2022; Liu et al. 2022; Özogul et al. 2022).

While numerous studies have explored the application of emulsion‐encapsulated essential oils in food preservation, the present study introduces a distinct and systematic comparative approach to address this knowledge gap. The unique advantage of this work lies in the strategic, side‐by‐side evaluation of two underutilized Iranian endemic plants, *S. bachtiarica* and *N. binaludensis*, within a single edible coating matrix. Furthermore, this study is the first to our knowledge to directly compare the efficacy of these essential oils in both their free and nanoemulsion forms when incorporated into a chitosan–gelatin (CG) composite coating. This design is critical, as it allows for the quantitative assessment of the added value provided by nanoencapsulation—specifically, enhanced stability, controlled release of bioactive compounds, and improved antimicrobial efficacy—against the benchmark of their free‐form counterparts. The selection of the CG blend is itself a synergistic innovation, combining chitosan's potent antimicrobial activity (Lv et al. 2023) with gelatin's superior oxygen barrier and mechanical properties (Erceg et al. 2023) to create an optimized delivery vehicle. By systematically comparing two endemic species and two delivery systems, this research provides a data‐driven rationale for selecting the most effective natural preservative strategy for meat products.

Beyond beef burgers, this coating system has potential applications in other high‐moisture muscle foods because the preservation mechanisms targeted in this study are common across these products. Moreover, the ultrasonication process and food‐grade biopolymer components used are compatible with industrial‐scale production, supporting the practical applicability of the proposed coating system (Gorzin et al. 2026; Özogul et al. 2022).

Therefore, the aim of this study was to investigate the effect of chitosan‐gelatin‐based composite edible coatings enriched with *S. bachtiarica* and *N. binaludensis* essential oils in free and nanoemulsion forms on the physicochemical, microbial, and sensory properties of beef burgers during refrigerated storage.

## 2 Materials and Methods

### 2.1 Materials

The aerial parts of the *S. Bachtiarica* plant from the city of Kord (Iran) and the *N. binaludensis* plant from the Binalod mountains in Khorasan province (Iran) were obtained. Bacterial strains of *Staphylococcus aureus* (PTCC1431), *Listeria monocytogenes* (ATCC19118), and *Escherichia coli* O157: H7 (ATCC35218) were prepared from the Food Science and Technology Department of Tehran University (Iran). Culture medium and chemicals were purchased from Merck Co. (Germany). Gelatin and chitosan (50,000–190,000 Da) were purchased from Sigma‐Aldrich (Canada).

### 2.2 Preparation of *S. bachtiarica* and *N. binaludensis* Essential Oils (NE)

The essential oils were extracted from the dried, powdered plants using a Clevenger apparatus. The aerial parts of both plants were washed with distilled water to remove surface impurities and dried under shade at ambient temperature (25°C ± 2°C) in a well‐ventilated environment until a constant weight was achieved. The dried materials were then ground using a laboratory grinder and passed through a 40‐mesh sieve to obtain a homogeneous powder prior to hydrodistillation. For each plant, 50 g of powder was combined with 500 mL of distilled water in a distillation flask and hydrodistilled for 4 h. The resulting essential oils were then dehydrated using anhydrous sodium sulfate before being stored in dark glass vials at refrigerated temperatures for subsequent analysis (Rabiei et al. 2022).

The phytochemical composition of the essential oils was determined by gas chromatography–mass spectrometry (GC–MS) (Agilent Technologies‐5975C‐MS, 7890A‐GC, USA) equipped with a 30 m × 0.25 mm capillary column (film thickness 0.25 μm) and a flame ionization detector (FID). The injector temperature was 250°C, helium was used as the carrier gas at 1 mL/min, and the injection volume was 1 μL. The oven temperature was programmed from 60°C to 300°C at 3°C/min (Erceg et al. 2023).

### 2.3 Preparation of Essential Oil Nanoemulsions

In this study, SEN and NEN nanoemulsions were prepared as oil‐in‐water (O/W) emulsions according to the method described by Hashemi Gahruie et al. (2018) with slight modifications. An aqueous solution containing 4.5% (w/w) Tween 80 in double‐distilled water was stirred for 30 min. SE/NE at a level of 6% (w/w) was gradually added to the aqueous phase and stirred for 30 min. Finally, using probe ultrasound (SONOPULS HD‐4200, Bandelin Con., Germany) at a temperature of 15°C, with a frequency of 40 kHz and a power of 100 for 20 min, the particle size of the formed emulsions was reduced and converted into a nanoemulsion form.

#### 2.3.1 Measurement of Average Particle Size, Polydispersity Index (PDI) and Zeta Potential

Determination of particle size and PDI of nanoemulsions was carried out at room temperature using dynamic light scattering (DLS) and Zetasizer (Sz 100, Horiba, Japan). Before testing, samples were diluted 100 times with deionized water (Lv et al. 2023).

#### 2.3.2 Morphology Study

The morphology of nanoemulsions was studied using a fluorescence microscope (Lionheart FX, Biotek Co., USA) equipped with a digital camera to confirm the successful preparation of these nanoemulsions.

#### 2.3.3 FTIR Spectrum Study

The FTIR spectra of free essential oils and their nanoemulsions were determined by FTIR spectrophotometer (Bruker, IFS‐48, Germany) at a wavelength of 400 to 4000/cm to investigate the functional groups and existing bands.

#### 2.3.4 Measurement of Total Phenol Content and Antioxidant Activity

The total phenol content (TPC) of free essential oils and their nanoemulsions was determined by the Folin–Ciocalteu method. 10% Folin–Ciocalteu solution was added to 0.5 mL of each sample and stirred well. After 6 min, 2 mL of sodium carbonate (7.5%) was added. After keeping for 30 min at room temperature and in the dark, the absorbance of the mixture was measured at a wavelength of 765 nm. To determine the TPC, the gallic acid standard curve was used (Gorzin et al. 2024).

To investigate the antioxidant activity of free essential oils and their nanoemulsions, the DPPH (2,2‐diphenyl‐1‐picrylhydrazyl) radical scavenging method was used. In this way, 4 mL of ethanolic DPPH (CAS No: 1898‐66‐4, Sigma‐Aldrich, Canada) solution (0.1 mmol/L) was added to each sample (0.2 mL), and then 8 mL of distilled water was added to the mixture, and after 30 min, the absorption of the mixture was recorded by UV–Vis spectrophotometer at 517 nm (*A*_s_). The control sample included distilled water and DPPH solution in the same amount used in the sample solution, and only the sample was not used in it (*A*_c_). Through the following equation (Equation 1), the antioxidant activity of essential oils and nanoemulsions was obtained in terms of the percentage of DPPH radical scavenging (Gorzin et al. 2024).

(1) $$\text{DPPH}\;\left(\%\right)=1-\frac{As}{Ac}\times 100$$

#### 2.3.5 Antimicrobial Activity Evaluation

To evaluate the antimicrobial activity of free essential oils and their essential oil nanoemulsions against *S. aureus* , *E. coli* , and *L. monocytogenes* , the agar disk diffusion method was used and the diameter of the growth inhibition zone against the target microorganisms was measured. The minimum inhibitory concentration (MIC) and minimum bactericidal concentration (MBC) values were also determined by the microdilution method (Özogul et al. 2022). To determine the diameter of the zone of bacterial inhibition, first 100 μL of each bacterial suspension (8 log CFU/mL concentration) was spread on the surface of the agar culture medium, and then sterile discs (filter paper) with a diameter of 6 mm were immersed in the SE and NE and their nanoemulsions (50 μL) separately, and the discs were placed on the inoculated culture media. The plates were incubated for 18–24 h at 37°C, and then the diameter of the zone of inhibition formed around each disc was measured in millimeters.

To determine the MIC and MBC, the microbial strains were first cultured on nutrient broth culture medium at 37°C for 24 h, and then the concentration of the microbial suspension was adjusted to 6 log CFU/mL. Sage and oregano essential oils were poured separately into test tubes and then serially diluted using Mueller‐Hinton broth to reach different concentrations (0.39, 0.78, 1.56, 3.12, 6.25, 25, and 50 mg/mL). After that, 1 mL of each bacterial inoculum was added to the tubes containing the diluted essential oils and nanoemulsions. The combination of Mueller‐Hinton broth and bacterial suspensions was used as a control. The prepared tubes were incubated in a rotating incubator at 37°C for 18–24 h and then examined. The lowest concentration of each sample that inhibited bacterial growth was considered as the MIC. To determine the MBC, 5 μL of aliquots were removed from tubes where no bacterial growth was observed and plated on Mueller Hinton agar medium, and the prepared culture media were incubated at 37°C for 24 h. The lowest concentration at which no bacterial growth was observed was considered as the MBC.

### 2.4 Preparation of Coating Solutions Based on Chitosan‐Gelatin (CG)

To prepare coating solutions based on CG, first a 1% (w/v) solution of chitosan powder in acetic acid was prepared at room temperature. Then a 3% (w/v) solution of gelatin in water was prepared. Initially, the gelatin solution was stirred for 15 min at 7°C until completely dissolved and then heated for 30 min at 55°C. Then the chitosan and gelatin solutions were mixed in equal proportions and stirred for 10 min at 60°C. After that, SE and NE and their nanoemulsions were added to the cooled coating solution at a level of 1 (v/v) and stirred. Finally, the pH of the solutions was adjusted to about 6.5 using sodium bicarbonate (Dzah et al. 2020).

### 2.5 Preparation of Coated Burger Treatments

The preparation of coated beef burgers was carried out according to the method described by Ashrafi et al. (2024) with slight modifications. Initially, fresh beef was ground using a meat grinder (whole diameter 3–5 mm). The hamburger formulation was as follows: 61.5% meat, 24% onion, 1.1% salt, 0.1% red pepper, 8% breadcrumbs, 5% wheat flour, 0.16% garlic powder, 0.07% turmeric, and 0.07% nutmeg. Three independent batches of burgers were prepared on different occasions, and all coating treatments were independently applied to each batch. Each analytical determination was subsequently performed using these independent biological replicates.

After mixing the formulation ingredients, burgers were formed using a mold with a diameter of 10 cm and a height of approximately 1 cm (approximately 80 g). The film‐forming solution consisted entirely of food‐grade, edible components: chitosan (1% w/v), gelatin (3% w/v), and essential oils (1% v/v) either in free or nanoemulsion form, all dissolved in 1% (v/v) acetic acid solution. All components are generally recognized as safe (GRAS) and approved for food contact applications (Abedinia et al. 2018; Erceg et al. 2023). Then, the burgers were immersed in CG‐based coating solutions for 2 min, which resulted in the formation of a thin, uniform surface layer (~0.3 mm thickness) without significant penetration into the burger interior, as the coating solution remained primarily on the surface due to the protein‐rich matrix of the burger acting as a barrier (Ashrafi et al. 2024), and after being removed from the solution and placed on mesh trays (for 2 min), the burgers were placed in polyethylene bags and stored at refrigerator (4°C ± 1°C) temperature for 12 days and tested every 4 days.

#### 2.5.1 pH Measurement

The pH of the burgers was calibrated using a pH meter (HM‐20S, Japan) and measured at 20°C. To prepare the samples, 10 g of each burger was mixed with 90 mL of water in a beaker using a glass rod (Homayonpour et al. 2021).

#### 2.5.2 Measurement of Total Volatile Basic of Nitrogen (TVB‐N)

Ten grams of Burgers were poured into a Kjeldahl flask, 2 g of magnesium oxide and 300 mL of distilled water were added. The resulting mixture was boiled by micro‐Kjeldahl for 25 min and the distilled liquid was collected. 25 mL of 2% (v/v) butyric acid solution and methylene blue and methylene red indicators were added to the resulting distilled liquid. The mixture was finally titrated with 0.1 N sulfuric acid solution and the TVB‐N content of the samples was obtained by multiplying the volume of sulfuric acid consumed by 14 and reported in mg N/100 g (Lv et al. 2023).

#### 2.5.3 Color Analysis

The color indices of the burgers, including L* (lightness), b* (yellow‐blue), and a* (red‐green), were assessed using a calorimeter (Minolta, China), and the overall color differences (Δ*E*) of the burgers compared to the first day was also evaluated using the below equation (Equation 2) (Fernandes et al. 2017).

(2) $$\Delta E=\sqrt{∆L^{2}+∆a^{2}+∆b^{2}}$$

#### 2.5.4 Microbial Analysis

Total counts of viable bacteria (VMBC) (plate count agar culture medium, incubation time and temperature of 48 h and 37°C, respectively), psychrophilic bacteria (PBC) (plate count agar culture medium, incubation time and temperature of 10 days and 7°C, respectively), and lactic acid bacteria (LAB) (MRS agar culture medium, incubation time and temperature of 72 h and 25°C, respectively) were performed in burgers using the method described by Keykhosravy et al. (2020). Enumeration of coliforms (Violet red bile dextrose agar (VRBA) culture medium, incubation time and temperature 24 h and 35°C, respectively) and molds and yeasts (Rose Bengal chloramphenicol agar culture medium, incubation time and temperature 3–5 days and 25°C, respectively) was also performed according to the method described by Farokhzad et al. (2023).

#### 2.5.5 Sensory Evaluation

Evaluation of the sensory characteristics of the burgers, including texture, flavor, odor, color, and overall acceptance, was performed by 20 panelists using a 5‐point hedonic test (7 = excellent, 4 = average, and 1 = very bad), which was conducted in individual booths under standardized lighting conditions (Bahmanyar et al. 2021).

The burgers were cooked in an oil‐free fryer (150°C) for approximately 8 min (until the center of the burger reached approximately 72°C) and, after cooling to room temperature (25°C ± 2°C), were provided to the evaluators on white, coded disposable plastic plates along with an evaluation form (Bahmanyar et al. 2021). Panelists consumed the cooked burger samples during the sensory evaluation. Accordingly, the attribute “flavor” refers to the perceived taste and overall flavor experienced during consumption, whereas odor and color were assessed independently before tasting. A mean overall acceptability score of less than 4.0, which corresponds to the “average” threshold on the hedonic scale, was considered the practical limit of consumer acceptance. The storage study was concluded on day 12 when the control samples fell below this threshold, while the treated samples maintained scores above it, thus demonstrating the preservative efficacy of the active coatings.

### 2.6 Statistical Analysis of Data

A completely randomized experimental design was used in this study. All tests were repeated three times and the statistical analysis of the obtained data was performed using SPSS 22.0 software and ANOVA: analysis of variance. Statistically significant differences between the treatments were examined at the 95% probability level (*p* < 0.05) by Duncan's multiple range test.

## 3 Results and Discussion

### 3.1 Phytochemical Composition of Essential Oils

The phytochemical composition of SE and NE was analyzed using gas chromatography–mass spectroscopy (GC–MS) and the results are presented in Tables 1 and 2, respectively. As can be seen in the tables, 37 and 50 phytochemical compounds were identified in the SE and NE, respectively. In SE, carvacrol (43.21%), thymol (21.82%), para‐cymene (11.43%), and γ‐terpinene (6.95%) were the main compounds and accounted for the largest share of the phytochemical compounds of this essential oil, while in NE, the largest share was 1,8‐cineole (29.30%), nepthalate (13.39%), germacerene D (5.36%), carvone (5.23%), α‐terpineol (4.25%), and piperitinone oxide (3.16%), respectively, and other compounds were also present in smaller amounts. Fathimoghaddam et al. (2020) reported carvacrol (31.25%), thymol (23.50%), and ocimene (13.87%) as the important compounds of this essential oil in the identification of phytochemical compounds of SE. In another study, carvacrol (59.8%), thymol (10.3%), *p*‐cymene (8.7%), γ‐terpinene (5.87%), linalool (2.35%), and β‐caryophyllene (2.21%) were identified as the main phytochemical compounds of SE (Pirastehfard et al. 2021). In a study conducted by Mohammadpour et al. (2013), 8,1‐cineole (68.31%), α‐terpineol (5.24%), and β‐pinene (4.74%) were the main phytochemical compounds identified in NE. Hashemi Moghaddam et al. (2020) also identified 1,8‐cineole and nepetalactone as the main phytochemical compounds of NE. In another study, 1,8‐cineole, nephthalactone, D‐germacerene, β‐pinene, and caryophyllene oxide were the main compounds of NE (Amirzadeh et al. 2022).

**TABLE 1:** Phytochemical composition of SE.

No.	Tx	RTn	RI	Amounts (%)	Compounds
1	5.423	4.7	927	1.183	α‐Thujene
2	5.511	4.7	930	1.436	α‐Pinene
3	6.107	4.7	953	0.128	Camphene
4	6.642	4.7	973	0.031	Sabinene
5	6.754	4.7	977	1.052	β‐Pinene
6	7.103	4.7	990	1.823	Myrcene
7	7.457	7.368	1002	0.296	α‐Phellandrene
8	7.64	7.368	1008	0.107	δ‐3‐Carene
9	7.864	7.368	1014	2.154	α‐Terpinene
**10**	**8.28**	**7.368**	**1026**	**11.43**	***p*‐cymene**
11	8.331	7.368	1027	0.917	Limonene
12	8.392	7.368	1029	0.19	1,8‐Cineole
13	8.645	7.368	1036	0.029	(Z)‐ β‐Ocimene
14	9.115	7.368	1049	0.129	(E)‐ β‐Ocimene
**15**	**9.506**	**7.368**	**1060**	**6.954**	**γ‐Terpinene**
16	9.868	7.368	1070	0.097	*cis*‐Sabinene hydrate
17	10.499	7.368	1088	0.248	Terpinolene
18	10.745	7.368	1095	0.058	Linalool
19	10.78	7.368	1096	0.073	*trans*‐Sabinene hydrate
21	14.01	10.93	1175	0.449	Terpinen‐4‐ol
22	14.634	10.93	1190	0.127	α‐Terpineol
23	16.505	15.05	1235	0.59	Carvacrol methyl ether
24	18.539	15.05	1283	1.087	(E)‐Anethol
**25**	**18.794**	**15.05**	**1289**	**21.824**	**Thymol**
**26**	**19.235**	**15.05**	**1299**	**43.213**	**Carvacrol**
27	22.243	19.26	1371	0.733	Carvacrol acetate
28	22.685	19.26	1382	0.172	Geranyl acetate
29	24.067	23.44	1416	0.47	(E)‐Caryophyllene
30	24.945	23.44	1437	0.198	Aromadendrene
31	26.068	23.44	1465	0.183	α‐Acoradiene
32	27.201	23.44	1493	0.095	Viridiflorene
33	27.601	27.48	1503	1.236	β‐Bisabolene
34	28.792	27.48	1534	0.128	(E)‐γ‐Bisabolene
35	29.815	27.48	1560	0.127	(E)‐Nerolidol
36	30.48	27.48	1577	0.35	Spathulenol
37	30.69	27.48	1583	0.716	Caryophyllene oxide

*Note:* Bold values indicate the major constituents identified in the essential oil based on their relative abundance.

**TABLE 2:** Phytochemical composition of NE.

No	Tx	RTn	RI	Amounts (%)	Compounds
1	5.247	4.7	921	0.012	Tricyclene
2	5.433	4.7	927	0.133	α‐Thujene
3	5.521	4.7	931	1.582	α‐Pinene
4	6.107	4.7	953	0.569	Camphene
5	6.563	4.7	970	1.433	Sabinene
6	6.675	4.7	974	2.217	β‐Pinene
7	7.105	4.7	990	0.874	Myrcene
8	7.152	4.7	992	0.262	3‐Octanol
9	7.472	7.368	1003	1.054	α‐Phellandrene
10	7.859	7.368	1014	0.117	α‐Terpinene
11	8.143	7.368	1022	1.255	*p*‐cymene
12	8.362	7.368	1028	7.061	Limonene
**13**	**8.438**	**7.368**	**1030**	**29.297**	**1,8‐Cineole**
14	8.54	7.368	1033	0.075	(Z)‐β‐Ocimene
15	8.995	7.368	1046	0.048	(E)‐β‐Ocimene
16	9.487	7.368	1059	0.495	γ‐Terpinene
17	9.893	7.368	1071	0.777	*p*‐Mentha‐3,8‐diene
18	10.283	7.368	1082	0.386	Terpinolene
19	10.901	7.368	1099	0.276	Isopentyl‐2‐methyl butanoate
20	11.358	10.93	1110	0.054	1‐Octen‐3‐yl acetate
21	11.831	10.93	1122	0.107	3‐Octanol acetate
22	12.441	10.93	1137	0.212	*trans*‐Pinocarveol
23	12.942	10.93	1149	1.663	*p*‐Menth‐3‐en‐8‐ol
24	13.045	10.93	1151	0.971	Menthone
25	13.452	10.93	1161	1.162	Menthofuran
26	13.562	10.93	1164	1.247	*neo*‐Menthol
27	13.599	10.93	1165	1.464	Borneol
28	13.889	10.93	1172	1.824	neoiso‐Isopulegol
29	13.97	10.93	1174	0.325	Menthol
30	14.331	10.93	1183	0.501	Dill ether
**31**	**14.602**	**10.93**	**1189**	**4.253**	**α‐Terpineol**
32	14.989	10.93	1198	0.899	*trans*‐Dihydro carvone
33	16.568	15.05	1236	0.973	Pulegone
**34**	**16.86**	**15.05**	**1243**	**5.233**	**Carvone**
35	17.224	15.05	1252	1.071	Piperitone
36	17.88	15.05	1267	0.354	Unknown
37	18.559	15.05	1283	0.69	Bornyl acetate
**38**	**20.929**	**19.26**	**1354**	**13.393**	**4a‐α,7‐α,7a‐α‐Nepetalacton**
**39**	**22.025**	**19.26**	**1362**	**3.177**	**Piperitenone oxide**
40	22.76	19.26	1384	0.272	β‐Bourbonene
41	23.998	23.44	1414	1.714	(E)‐Caryophyllene
42	25.63	23.44	1454	0.303	α‐Humulene
**43**	**26.837**	**23.44**	**1484**	**5.357**	**Germacrene D**
44	27.357	23.44	1497	0.199	Bicyclogermacrene
45	27.948	27.48	1512	0.068	γ‐Cadinene
46	28.184	27.48	1518	0.143	δ‐Cadinene
47	29.801	27.48	1560	0.149	Carvotacetone acetate
48	30.498	27.48	1578	0.088	Spathulenol
49	30.691	27.48	1583	0.231	Caryophyllene oxide
50	32.112	31.37	1620	0.302	Dill apiole

*Note:* Bold values indicate the major constituents identified in the essential oil based on their relative abundance.

### 3.2 Characteristics of Essential Oils Nanoemulsions

#### 3.2.1 Average Particle Size, PDI Index and Thermodynamic Stability

**FIGURE 1:**
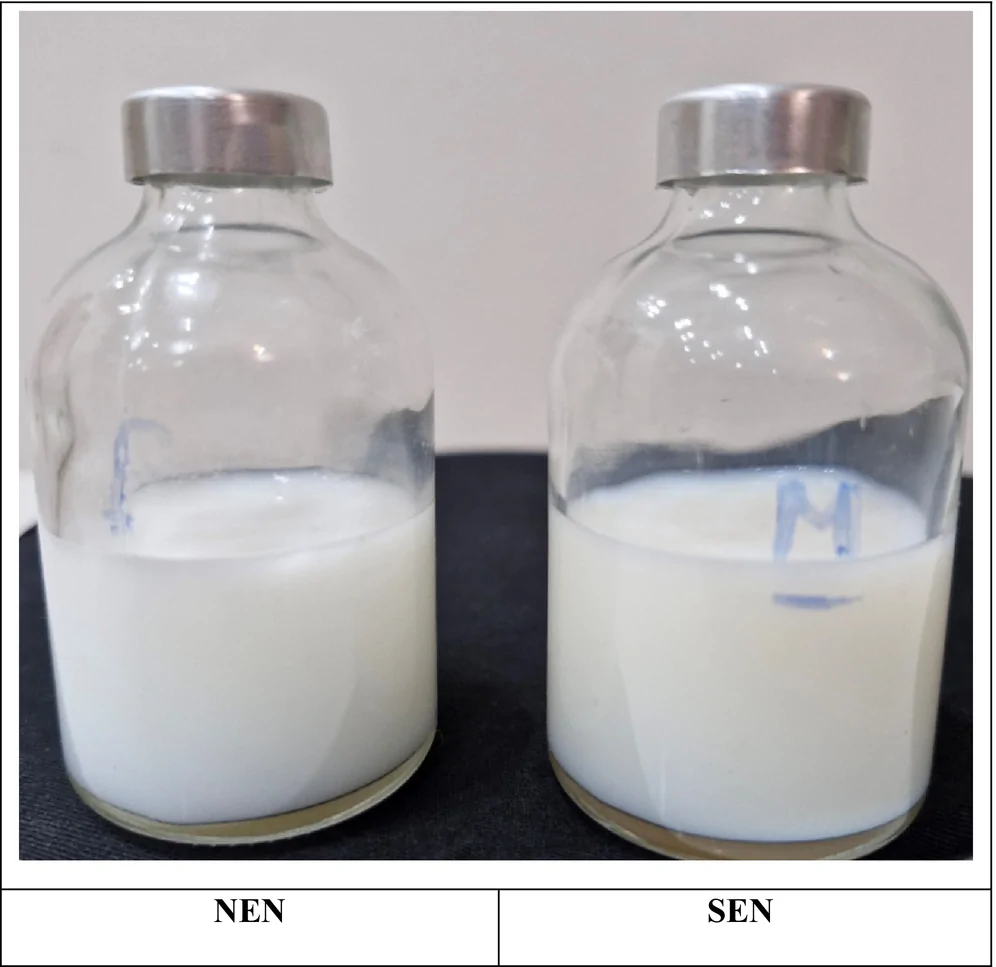
Images of SEN and NEN after 2 weeks of storage at room temperature.

The particle size obtained in this study indicates the desired efficiency of the production and homogenization process used to form nanoemulsions. The average particle size of SEN and NEN nanoemulsions was 122.4 and 65.6 nm, respectively (Table 3), and in general, both nanoemulsions had a particle size in the nanometer and small range. Research has shown that when Tween 80 is used as a surfactant to produce nanoemulsions, small‐sized particles are produced because this surfactant has a small size and, since it is rapidly absorbed on the droplet surfaces, it shows a better ability to minimize the size of the produced particles compared to biopolymers (Noori et al. 2018). The PDI is used to evaluate the dispersion or heterogeneity of particle size of a sample and its numerical value ranges from 0 (completely uniform particles) to 1 (particles with different droplet sizes); therefore, the lower the PDI of the produced particles, the more regular the particle size distribution (Özogul et al. 2022). In general, a low PDI indicates the favorable ability of the ultrasonication method to form nanoemulsions with a more uniform particle size distribution (Noori et al. 2018). Since the PDI of SEN (0.286) and NEN (0.391) nanoemulsions was lower than 0.5, it indicates good dispersion of oil droplets in the system. The PDI of nanoemulsions is related to the concentration of surfactant used, the amount of aqueous phase used, and the conditions of emulsification by ultrasound (Chu et al. 2020). To assess storage stability, the particle size, PDI, and zeta potential of SEN and NEN nanoemulsions were monitored over 14 days of storage at 25°C ± 1°C. As shown in Table 3, the mean particle size of SEN and NEN increased marginally from 122.4 ± 3.8 to 135.7 ± 4.2 nm and from 65.6 ± 2.1 to 78.3 ± 3.4 nm, respectively, after 14 days. The PDI values remained below 0.5 for both nanoemulsions throughout storage (SEN: 0.286 ± 0.021 to 0.319 ± 0.027; NEN: 0.391 ± 0.033 to 0.428 ± 0.038), indicating a relatively uniform particle size distribution. The zeta potential values were measured as −32.4 ± 2.1 mV for SEN and −26.7 ± 1.9 mV for NEN immediately after preparation, which decreased slightly to −28.6 ± 2.3 and −23.5 ± 2.0 mV, respectively, after 14 days. These values, remaining above the critical threshold of ±25 mV, suggest that electrostatic repulsion was sufficient to maintain colloidal stability and prevent significant aggregation (Chu et al. 2020; Özogul et al. 2022). In Figure 1, images of SEN and NEN nanoemulsions are shown after 2 weeks of storage at room temperature, and as can be seen in the figure, no two‐phase formation occurred in these nanoemulsions during the storage period, which is consistent with the quantitative stability parameters presented in Table 3. Özogul et al. (2022) obtained the average particle size of Laurel nanoemulsion as 247.52 nm, and these researchers also stated that the produced nanoemulsion had good thermodynamic stability for more than 2 weeks of storage at room temperature. Pirastehfard et al. (2021) prepared nanoemulsions of canola oil combined with SEN and reported the particle size and PDI of the produced nanoemulsion as 243 and 0.293 nm, respectively. These researchers also stated that this nanoemulsion had favorable thermodynamic stability. In another study, the average particle diameter and PDI of the essential oil of *Satureja sahendica Bornm* were obtained as 473.2 nm and 0.182, respectively, and these researchers also observed favorable stability of the produced nanoemulsion for at least 14 days at room temperature (Gholamhosseinpour et al. 2023).

**TABLE 3:** Mean particle size, polydispersity index, zeta potential, TPC and antioxidant activity of SE and NE and their nanoemulsions.

Samples	*Z*‐average (nm)	PDI	Zeta potential (mV)	TPC (mg GAE/g)	DPPH (%)
SE	—	—	—	33.49 ± 0.55^a^	74.62 ± 0.95^a^
SEN	122.4 ± 7.3^a^	0.286 ± 0.019^b^	−26.7 ± 1.4^b^	31.87 ± 0.43^b^	75.93 ± 0.84^a^
NE	—	—	—	23.61 ± 0.28^c^	65.41 ± 0.87^b^
NEN	65.6 ± 5.9^b^	0.391 ± 0.026^a^	−38.2 ± 5.9^a^	22.37 ± 0.40^d^	67.10 ± 1.16^b^

*Note:* Values represent mean (*n* = 3) ± SD. Different letters in each column represent significant difference at 5% level of probability among samples.

#### 3.2.2 Zeta Potential

Zeta potential is an index for displaying the electrical charge of the surface of particles, which is effective in evaluating the stability of produced particles in colloidal systems. A higher absolute zeta potential value generally enhances electrostatic repulsion between droplets and reduces aggregation during storage (Homayonpour et al. 2021). The zeta potential values of SEN and NEN nanoemulsions were −26.7 and −38.2 mV, respectively, indicating acceptable physical stability of the prepared systems. Although Tween 80 is a nonionic surfactant and does not directly generate surface charge, it can contribute to nanoemulsion stability by forming a hydrated interfacial layer around oil droplets and preventing droplet coalescence through steric stabilization (Ziani et al. 2011). Therefore, the negative zeta potential observed in this study is mainly attributed to the adsorption and ionization of surface‐active phytochemical constituents present in the essential oils, including compounds containing hydroxyl and carboxyl functional groups, rather than direct charge contribution from Tween 80. In general, absolute zeta potential values above 30 mV are considered indicative of enhanced resistance against aggregation in colloidal dispersions.

Acevedo‐Fani et al. (2015) also reported negative surface charges for essential oil nanoemulsions and attributed this behavior to ionizable groups of phytochemical compounds. Similarly, Das et al. (2024) reported a negative zeta potential (~30 mV) for clove essential oil nanoemulsions.

#### 3.2.3 Morphology

**FIGURE 2:**
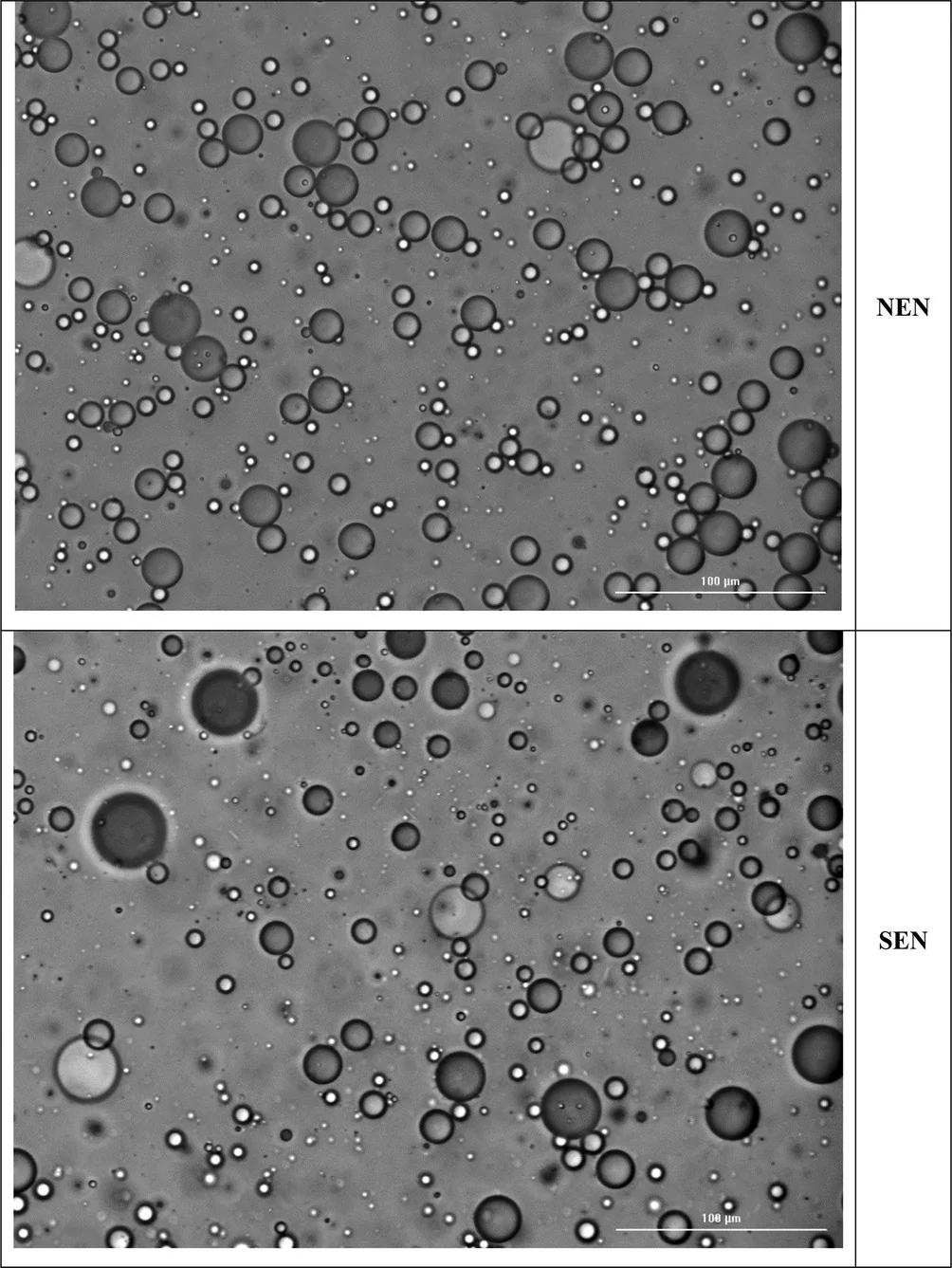
Fluorescence microscope images of SEN and NEN.

The morphology of SEN and NEN was examined by optical microscopy and the resulting images are shown in Figure 2. As expected, the droplets of the nanoemulsions produced in this study were globular (spherical) with completely smooth surfaces and no holes or wrinkles were observed on their surfaces. No aggregation was observed among the particles, which is consistent with the results related to the high zeta potential of these particles and their favorable stability in colloidal systems. The formed oil droplets were mostly small and uniform in size, but a few droplets with larger sizes were also observed among them, and this difference in particle size in the SEN was greater than that in the NEN, which was consistent with the results related to the PDI index values of these nanoemulsions, because the higher the PDI index of a nanoemulsion, the greater the difference in particle size. The presence of larger droplets indicates that some droplets were not properly reduced during the homogenization process. In the study of Chu et al. (2020), according to the fluorescence microscope image, the produced cinnamon essential oil nanoemulsions had a uniform dispersion of oil droplets. Das et al. (2024) also showed a spherical shape with smooth surfaces of the clove essential oil nanoemulsion. Liu et al. (2022) also reported a spherical shape and good distribution of the lemon essential oil nanoemulsion.

#### 3.2.4 FTIR Spectra

**FIGURE 3:**
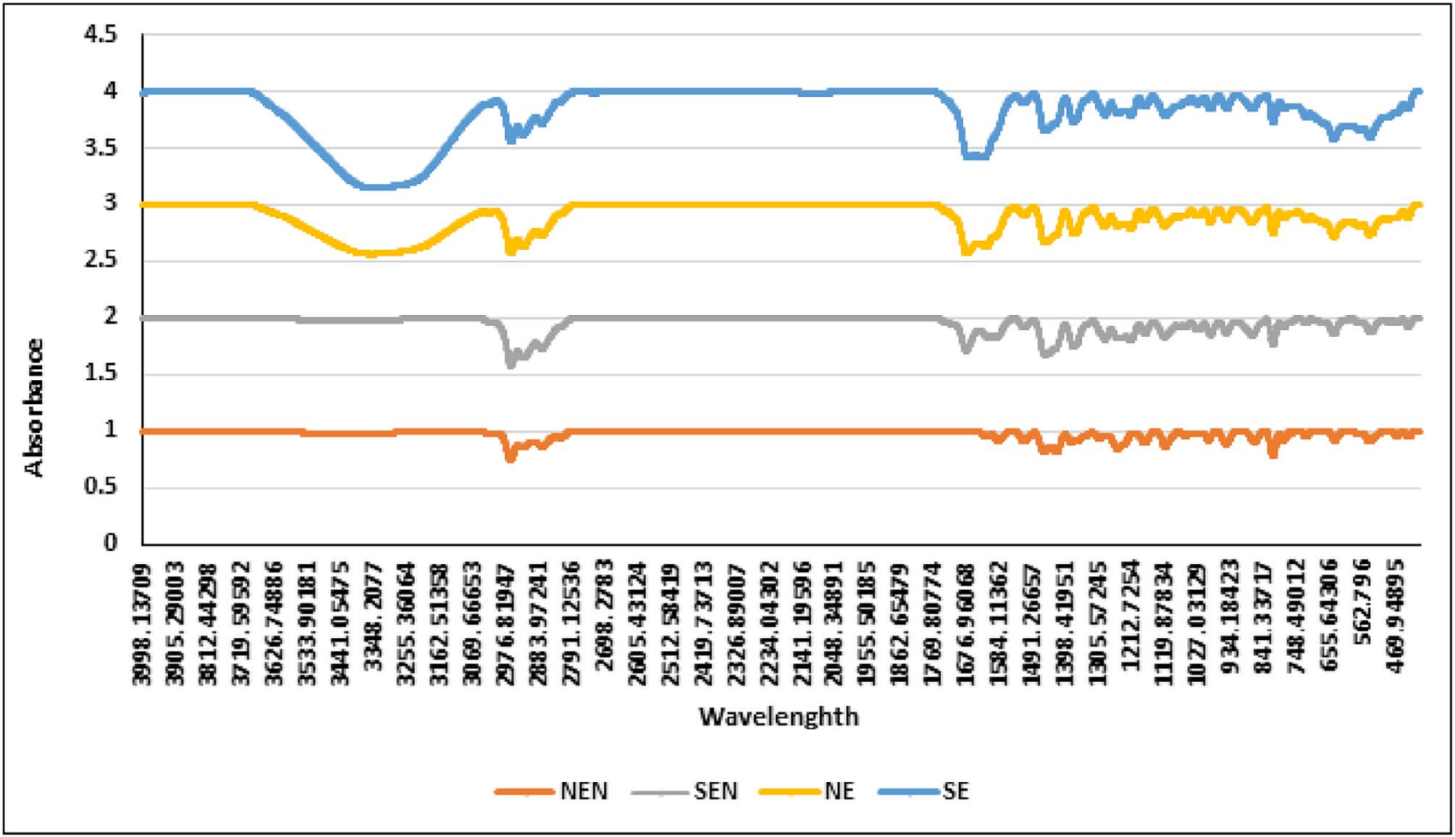
FTIR spectra of SE and NE and their nanoemulsions.

FTIR spectroscopy is a method used to detect functional groups in the chemical structures of compounds and shows the presence of specific compounds and bands. The functional groups of SE and NE and their nanoemulsions were investigated by FTIR spectroscopy and the resulting spectra are presented in Figure 3. In the FTIR spectra of the essential oils, a broad and large peak was observed as an indicator peak in the region of 3300/cm, which is related to the hydroxyl group (O—H). There were four peaks in the region of 2128–2958/cm, which are related to the stretching vibrations of the C=C—C groups of volatile compounds and the symmetric and asymmetric stretching of the C—H bond (Cebi et al. 2021). A series of peaks were observed in the region 1674–1369/cm that are related to aromatic rings and these peaks were also observed in the study conducted by Elsherif and Talaat Al Shrief (2021). The peaks in the region 1300–1200/cm also indicate the stretching vibrations of the C—O and C—O—C bonds of phenolic compounds, and the peaks in the region 1100–1000/cm are also related to the vibrations of hydroxyl groups (Cebi et al. 2021). The peaks in the region below 1000/cm can also be related to the stretching vibrations of the C—H bond (Tavares and Noreña 2020). Peaks related to essential oils were also observed in the study conducted by Liu and Liu (2020), and these researchers also showed that in the thyme essential oil nanoemulsion, the peaks related to the essential oil were weakened and the peak related to the hydroxyl group was not observed in the FTIR spectrum of the essential oil nanoemulsion, and these researchers also found that the thyme essential oil was physically located in the nanoemulsion structure and did not establish a chemical bond. Gharenaghadeh et al. (2017) also found in agreement that in the production of the Salvia multicaulis essential oil nanoemulsion, the peaks related to the essential oil were either eliminated in the essential oil nanoemulsion peak or were weakly present, and these researchers also reported physical bonding between the essential oil and the nanoemulsion.

#### 3.2.5 TPC and Antioxidant Activity

The TPC of SE and NE, both free and nanoemulsions, was investigated by the standard Folin‐Ciocaltio method and their antioxidant activity was investigated by the DPPH radical scavenging method and the results are shown in Table 3. In general, the TPC of SE in both forms (31.4879–33.49 mg GAE/g) was higher than the TPC of NE (22.37–23.61 mg GAE/g) and the encapsulation process in nanoemulsions significantly reduced the TPC of essential oils (*p* < 0.05). High TPC and antioxidant activity of SE have also been reported by Fathimoghaddam et al. (2020). Mahalleh et al. (2021) also demonstrated considerable antioxidant activity of encapsulated plant extracts.

Despite the reduction in measured TPC after nanoemulsification, the DPPH radical scavenging activity of encapsulated essential oils was comparable to or slightly higher than that of free oils. This observation suggests that antioxidant capacity cannot be explained solely by total phenolic concentration, as the Folin–Ciocalteu assay mainly reflects extractable reducing compounds, whereas DPPH activity depends on the accessibility and reactivity of antioxidant molecules during radical neutralization. The improved antioxidant response after nanoemulsification may be related to enhanced dispersion of oil droplets, increased interfacial area, and improved accessibility of bioactive compounds at the oil–water interface. Furthermore, Tween 80 has been reported to exhibit antioxidant‐related activity and may contribute partially to the measured radical scavenging capacity of surfactant‐stabilized systems (Pérez‐Rosés et al. 2015). Similar observations have been reported for Tween 80‐based encapsulation systems, where surfactant‐assisted stabilization contributed to antioxidant retention during processing (Sarabandi and Jafari 2020). The antioxidant activity of SE and NE was in the range of 74.62%–75.93% and 65.41%–67.10%, respectively. Liu et al. (2022) reported higher DPPH radical scavenging activity for lemon essential oil nanoemulsions compared with free essential oil. Similarly, Gholamhosseinpour et al. (2023) reported enhanced antioxidant activity after nanoemulsification of Satureja sahendica essential oil.

#### 3.2.6 Antimicrobial Activity

In this study, the agar disk diffusion method (determination of the diameter of the zone of inhibition) and the microdilution method (determination of MIC and MBC values) were used to investigate the antimicrobial activity of SE and NE in both free and nanoemulsion forms. The results of the investigation of the antimicrobial activity of essential oils by microdilution in the form of MIC and MBC values are presented in Table 4. This test also shows that, with the exception of *E. coli* , the MIC values of the nanoemulsions of the essential oils were lower than their free forms. In the case of *E. coli* , the MIC values of the free and nanoemulsions of the essential oils were similar. In this test, the antimicrobial activity of the essential oils against Gram‐positive bacteria was higher than that of Gram‐negative bacteria because they had lower MIC values. SE also had higher antimicrobial activity than NE and showed higher growth inhibitory effect at lower concentrations. For all bacteria studied in this study, MBC values were higher than MIC values, and therefore higher amounts of essential oil were required to kill bacteria compared to inhibit their growth.

**TABLE 4:** Antibacterial activity of free and nano‐encapsulated BE and AE against two major pathogenic bacteria.

Bacteria	SE	SEN	NE	NEN
Diameters of inhibitory zone (mm)				
*S. aureus*	11.38 ± 0.25^c^	15.07 ± 0.36^a^	9.45 ± 0.31^d^	12.35 ± 0.52^b^
*L. monocytogenes*	12.02 ± 0.32^b^	14.35 ± 0.22^a^	10.11 ± 0.19^c^	12.24 ± 0.15^b^
*E. coli O157:H7*	7.41 ± 0.18^b^	9.63 ± 0.15^a^	6.77 ± 0.14^d^	7.12 ± 0.09^c^
MIC (mg/mL)				
*S. aureus*	6.25	3.12	25	6.25
*L. monocytogenes*	6.25	3.12	12.5	6.25
*E. coli O157:H7*	12.5	12.5	25	25
MBC (mg/mL)				
*S. aureus*	12.5	6.25	50	25
*L. monocytogenes*	12.5	6.25	25	12.5
*E. coli O157:H7*	25	25	< 50	50

*Note:* Values represent mean ± SD. Different letters indicate significant difference among samples at 5% probability level.

**FIGURE 4:**
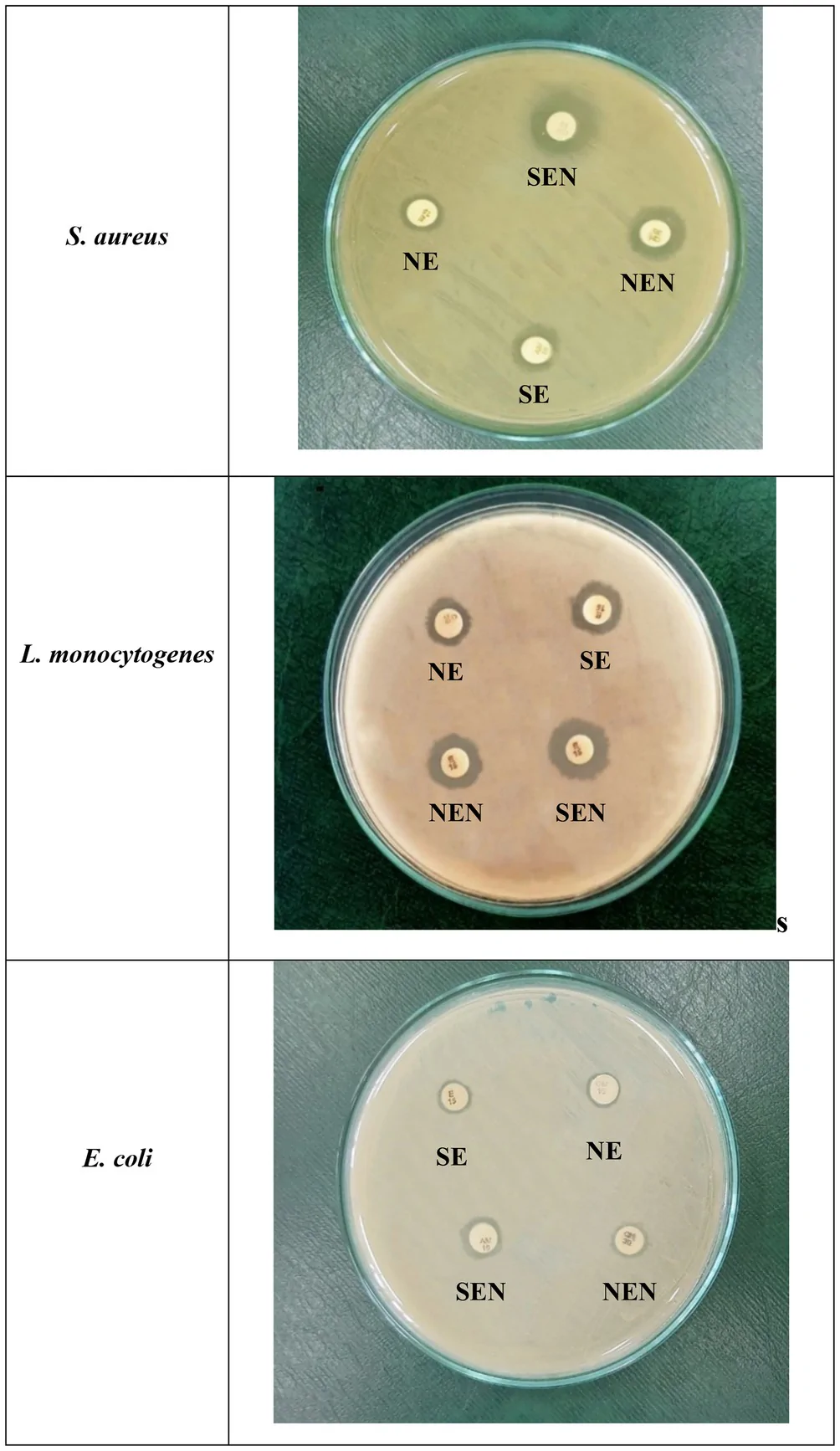
Images of the antimicrobial activity of SE and NE and their nanoemulsions against foodborne pathogens using the agar disk diffusion method.

The results of the antimicrobial activity study using the agar disk diffusion method are presented in Figure 4, and show that the antimicrobial activity of essential oils and their nanoemulsions against the gram‐positive bacteria *S. aureus* (9.45–15.07 mm) and *L. monocytogenes* (10.11–14.35 mm) was higher than the gram‐negative bacteria *E. coli* (6.77–9.63 mm). The higher antimicrobial activity of plant essential oils against Gram‐positive bacteria compared to Gram‐negative bacteria is related to the more complex cell wall of Gram‐negative bacteria, because the cell wall of these bacteria is composed of two layers, including a layer of lipopolysaccharides and a second layer of peptidoglycan, which acts as a complex barrier and reduces the absorption of bioactive compounds and reactive oxygen species into the bacterial cell (Russell 2003). These results were consistent with the findings of Özogul et al. (2022), and Gholamhosseinpour et al. (2023). SE generally showed higher antimicrobial activity than NE, and this difference is related to the different types and amounts of phytochemical and active compounds present in these two essential oils. Plant essential oils in general often have antibacterial activity, and the bioactive compounds in them (terpenoid compounds with hydrophobic nature) are able to interact with lipids in the bacterial cell membrane and disrupt the structure of the cell membrane, disrupting the conditions of cell homeostasis and increasing its permeability. Due to the increase in the permeability of the bacterial cell membrane, intracellular contents leak out and lead to cell death (Yazgan et al. 2019). Changes in fatty acids and phospholipids of the bacterial cell also disrupt RNA and DNA synthesis and cause cellular energy depletion. Essential oil compounds also lead to protein transport degradation (Özogul et al. 2022). Fathimoghaddam et al. (2020) also confirmed the significant antimicrobial activity of SEN. High antibacterial activity of SE against *E. coli* was also observed by Hashemi and Khodaei (2020). In a study conducted by Babadi et al. (2012), thymol and carvacrol were also introduced as responsible for the significant antibacterial activity of SE, which causes damage to the bacterial cell membrane and leads to bacterial death by efflux of intracellular ATP and potassium ions. Khaladgi (2018) also showed that *N. binaludensis Jamzad* extract showed antimicrobial activity against both Gram‐positive and Gram‐negative bacteria and mold. Amirzadeh et al. (2022) also reported the antibacterial activity of NE against *E. coli* . These researchers identified 1,8‐cineole and nepetalactone as the antimicrobial compounds of this essential oil. The results of the present study also showed that the nanoencapsulation process was able to improve the antibacterial activity of essential oils. During the production process of nanoemulsions, due to the reduction in particle size and increase in the surface area of the droplets, their penetration into the bacterial cell can occur faster, and thus the antimicrobial function of the active agents is faster and more effective (Noori et al. 2018). This process is also able to improve the physicochemical stability of essential oils and prevents evaporation and loss of essential oil (de Matos et al. 2019). Improved antimicrobial activity of plant essential oils after encapsulation has also been reported by previous researchers (Gholamhosseinpour et al. 2023; Lv et al. 2023).

### 3.3 Burger Tests

#### 3.3.1 pH

Table 5 shows an appearance of samples and the changes in the average pH values of coated burgers during a 12‐day storage period under refrigerated conditions. At the beginning of the storage period, the control sample had the highest pH (5.43) and coating the burgers with CG‐based solutions significantly reduced the pH of the burgers on this day to 5.37 (*p* < 0.05); however, the addition of essential oils did not show a significant effect. These observations are related to the use of acetic acid to prepare the coating solutions containing chitosan. During the storage period, the pH of the burgers gradually increased due to the growth of microorganisms and the secretion of protease enzymes and protein degradation, resulting in the production of volatile nitrogen compounds (*p* < 0.05). Due to the antimicrobial activity of the coatings used in this study, the intensity of the increase in pH of the coated burgers, especially with solutions enriched with SE and NE, significantly decreased compared to the control. At the end of the storage period, the control sample had the highest pH (6.07) and the coating without essential oil was next (5.67). Treatments coated with solutions enriched with essential oils, especially in nanoemulsion form, had significantly lower pH on the last day (*p* < 0.05), and the lowest pH on this day was observed in the burger coated with a solution containing SEN (5.43) and NEN (5.44). In line with these results, Lv et al. (2023) also observed that CG‐based edible coatings containing encapsulated clove extract were able to significantly reduce the rate of changes in the pH of pork during storage compared to the uncoated sample. Gaba et al. (2025) also significantly reduced the severity of pH changes in beef burgers during storage at refrigerated temperature using a chitosan‐based edible coating containing oregano and thyme essential oils. Farokhzad et al. (2023) also reported an increase in the pH of beef burgers during storage at refrigerated temperature and were able to reduce the severity of changes in the pH of burgers over time using a chitosan coating containing rosemary essential oil compared to the control.

**TABLE 5:** Photographs of the uncoated, coated burgers and comparison of pH, TVB‐N, peroxide, and TBA indexes of beef burgers during the storage period.

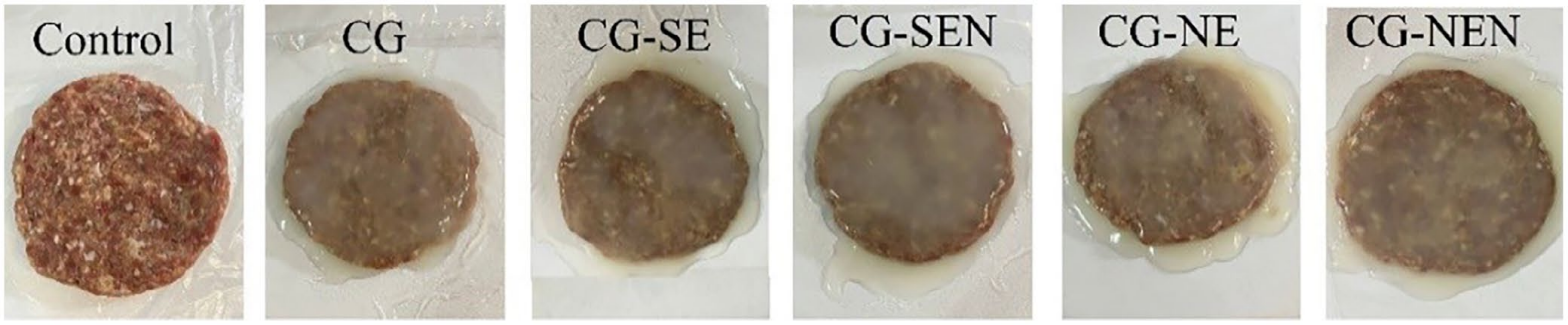			
Samples	Storage time (Day)	pH	TVB‐N (mg N/100 g)
Control	1	5.43 ± 0.02^D,a^	10.16 ± 0.18^D,a^
	4	5.59 ± 0.01^C,a^	18.94 ± 0.23^C,a^
	8	5.78 ± 0.01^B,a^	26.06 ± 0.31^B,a^
	12	6.07 ± 0.00^A,a^	31.62 ± 0.24^A,a^
CG	1	5.37 ± 0.01^D,b^	9.93 ± 0.15^D,a^
	4	5.40 ± 0.01^C,b^	14.10 ± 0.20^C,b^
	8	5.57 ± 0.02^B,b^	19.33 ± 0.13^B,b^
	12	5.67 ± 0.03^A,b^	22.86 ± 0.19^A,b^
CG‐SE	1	5.36 ± 0.02^C,b^	9.98 ± 0.24^D,a^
	4	5.37 ± 0.02^C,b,c^	11.47 ± 0.17^C,d^
	8	5.42 ± 0.01^B,d^	13.69 ± 0.15^B,e^
	12	5.46 ± 0.01^A,d^	15.97 ± 0.21^A,d^
CG‐SEN	1	5.36 ± 0.01^C,b^	9.95 ± 0.16^D,a^
	4	5.37 ± 0.01^b,c,c^	11.15 ± 0.32^C,d^
	8	5.39 ± 0.02^B,d^	13.24 ± 0.26^B,f^
	12	5.43 ± 0.01^A,e^	14.90 ± 0.10^A,e^
CG‐NE	1	5.38 ± 0.01^B,b^	10.04 ± 0.15^D,a^
	4	5.39 ± 0.01^B,b^	12.35 ± 0.20^C,c^
	8	5.46 ± 0.02^A,c^	14.90 ± 0.14^B,c^
	12	5.51 ± 0.03^A,c^	18.21 ± 0.36^A,c^
CG‐NEN	1	5.36 ± 0.02^C,b^	9.99 ± 0.21^D,a^
	4	5.37 ± 0.01^C,c^	11.73 ± 0.28^C,d^
	8	5.40 ± 0.01^B,d^	14.39 ± 0.19^B,d^
	12	5.44 ± 0.00^A,e^	16.42 ± 0.33^A,d^

*Note:* Values represent mean (*n* = 3) ± SD. Small and big different letters indicate significant difference among samples and storage period at 5% level of probability, respectively.

#### 3.3.2 TVB‐N

The trend of changes in TVB‐N values of coated burgers during a 12‐day storage period under refrigerated conditions can be seen in Table 5. At the beginning of the storage period, TVB‐N values of different burger treatments were in the range of 9.93–10.16 mg N/100 g, and coating burgers with CG‐based solutions on this day did not show a significant effect on the TVB‐N levels of burgers. During the storage period, due to the activity of microorganisms and the action of protease enzymes and protein degradation, as well as reactions such as amino acid deamination and nucleotide degradation (Tometri et al. 2020), the TVB‐N content of different burger treatments increased significantly (*p* < 0.05), but due to the antimicrobial activity of the coatings used in this study, the intensity of the increase in TVB‐N of burgers coated with active coatings containing essential oils decreased significantly compared to the control. At the end of the storage period, the control sample had the highest TVB‐N content (31.62 mg N/100 g), followed by the burger coated with CG solution without essential oils (23.86 mg N/100 g). Treatments coated with essential oil‐enriched solutions, especially in their nanoemulsion form, had significantly lower TVB‐N levels on the last day (*p* < 0.05), and the lowest TVB‐N levels on this day were observed in burgers coated with SEN‐containing solution (14.90 mg N/100 g). In line with these results, Hashemi, Adibi, et al. (2023) also observed that alginate coatings containing *Carum copticum* essential oil in free and encapsulated forms were able to reduce TVB‐N levels in fish burgers during storage, and these researchers found that during storage, TVB‐N levels in burgers coated with a coating containing encapsulated *C. copticum* essential oil were lower than those in the free form of this essential oil. The reduction in the production rate of TVB‐N in meat products using active edible coatings has also been reported by other researchers (Gaba et al. 2025; Isvand et al. 2024; Lv et al. 2023). The maximum TVB‐N level for meat and meat products is recommended to be 25 mg N/100 g (Farokhzad et al. 2023). Accordingly, the control sample had acceptable TVB‐N levels until the fourth day and the other treatments until the last day of refrigerated storage (day 12).

#### 3.3.3 Color

Color is one of the important quality parameters of meat and meat products that affects the final acceptance of these products by consumers. The color of different coated burger treatments was examined by calorimeter and the determined color indices are presented in Table 6. At the beginning of the storage period, coating burgers with CG‐based solutions on this day did not show a significant effect on the L*, a* and b* indices of burgers, and the values of these indices of different burger treatments were obtained in the range of 49.10–49.86, 12.66–12.91 and 8.34–8.62, respectively. During the storage period, the L* index of different burger treatments gradually decreased, but this decrease was statistically significant only in the control sample from the fourth day onwards and in the burger coated with CG solution without essential oil from the eighth day onwards (*p* < 0.05). Over time, a decrease in the a* index and an increase in the b* index of burgers were also observed. The use of active CG‐based coatings, especially coatings enriched with essential oils, was able to significantly reduce the rate of decrease in the intensity of the redness of burgers compared to the control. The results of the Δ*E* index study showed that on the fourth day of storage, the highest Δ*E* index was for the control sample (3.16), and there was no statistically significant difference between the Δ*E* index of burgers coated with CG‐based solutions on this day, and its level in these treatments was in the range of 0.23–0.67. Over time, the Δ*E* index of different burger treatments gradually increased, but the highest rate of increase was for the control sample. At the end of the storage period, the control sample had the highest Δ*E* index (8.85), and the burger coated with CG solution without essential oils was next (4.06). On this day, there was no statistically significant difference between the Δ*E* index of other treatments. One of the main reasons for color changes in meat products during storage is the oxidation of lipids, which causes discoloration in these products during storage. In general, secondary products formed during the oxidation process react with natural meat pigments (including oxymyoglobin) and as a result of this reaction, dark and brown pigments (including metmyoglobin) are produced. These pigments darken the meat color and reduce the intensity of redness of meat during storage (Kowalczyk et al. 2023). Chitosan used in coating solutions has metal chelating effects and thus reduces the intensity of meat pigment oxidation over time (Carrapiso et al. 2023). Edible coatings in general are able to reduce the intensity of pigment oxidation in meat over time by reducing available oxygen, thereby improving the color retention of these products (Farokhzad et al. 2023). In agreement with these results, Ghaderi‐Ghahfarokhi et al. (2017) also observed a significant efficacy of cinnamon essential oil encapsulated in chitosan in preserving the color of beef patties during storage under refrigerated conditions compared to the control. In another study, during the storage period at refrigerated temperature, the meat color darkened, which the researchers attributed to the formation of a dark brown color in this product during the storage period due to the production of metmyoglobin pigment, a gradual decrease in moisture, and a decrease in light refraction (Amiri et al. 2019). Carrapiso et al. (2023) also observed that during the storage period at refrigerated temperature, the intensity of the red color of pork burgers decreased, but the use of an edible coating based on chitosan containing olive leaf extract caused a decrease in its intensity.

**TABLE 6:** Comparison of color indices of beef burgers during the storage period.

Samples	Storage time (Day)	L*	a*	b*	Δ*E*
Control	1	49.10 ± 1.04^A,a^	12.91 ± 0.13^A,a^	8.34 ± 0.21^D,a^	—
	4	47.42 ± 1.17^A,a^	10.30 ± 0.21^B,b^	8.95 ± 0.26^C,a^	3.16 ± 0.30^C,a^
	8	44.93 ± 1.11^B,b^	8.15 ± 0.08^C,c^	9.74 ± 0.17^B,a^	6.48 ± 0.19^B,a^
	12	43.15 ± 0.89^B,b^	6.99 ± 0.16^D,c^	11.13 ± 0.19^A,a^	8.85 ± 0.47^A,a^
CG	1	49.36 ± 1.10^A,a^	12.79 ± 0.10^A,a^	8.55 ± 0.28^C,a^	—
	4	48.80 ± 0.86^A,a^	12.46 ± 0.19^B,a^	8.67 ± 0.19^C,a,b^	0.67 ± 0.28^C,b^
	8	47.91 ± 0.88^A,B,a^	11.38 ± 0.12^C,b^	9.10 ± 0.11^B,b^	2.10 ± 0.44^B,b^
	12	46.79 ± 1.02^B,a^	10.01 ± 0.23^D,b^	10.02 ± 0.24^A,b^	4.06 ± 0.36^A,b^
CG‐SE	1	49.52 ± 0.93^A,a^	12.85 ± 0.19^A,a^	8.62 ± 0.15^A,a^	—
	4	49.23 ± 1.13^A,a^	12.68 ± 0.15^A,B,a^	8.69 ± 0.22^A,a,b^	0.34 ± 0.19^C,b^
	8	48.57 ± 1.02^A,a^	12.30 ± 0.24^b,c,a^	8.75 ± 0.13^A,c^	1.11 ± 0.18^B,c^
	12	47.83 ± 1.23^A,a^	12.08 ± 0.11^C,a^	8.90 ± 0.19^A,c^	1.87 ± 0.32^A,c^
CG‐SEN	1	49.86 ± 0.86^A,a^	12.70 ± 0.16^A,a^	8.41 ± 0.24^A,a^	—
	4	49.51 ± 0.95^A,a^	12.61 ± 0.12^A,a^	8.52 ± 0.10^A,b^	0.27 ± 0.14^B,b^
	8	48.98 ± 0.89^A,a^	12.49 ± 0.29^A,B,a^	8.66 ± 0.16^A,c^	0.35 ± 0.20^B,d^
	12	48.10 ± 1.14^A,a^	12.13 ± 0.16^B,a^	8.84 ± 0.25^A,c^	1.88 ± 0.27^A,c^
CG‐NE	1	49.29 ± 1.15^A,a^	12.66 ± 0.20^A,a^	8.60 ± 0.11^B,a^	—
	4	48.75 ± 1.24^A,a^	12.53 ± 0.17^A,a^	8.68 ± 0.24^A,B,a,b^	0.56 ± 0.18^B,b^
	8	48.16 ± 1.07^A,a^	12.15 ± 0.10^B,a^	8.74 ± 0.21^A,B,c^	1.25 ± 0.32^B,c^
	12	47.41 ± 0.86^A,a^	11.72 ± 0.28^C,a^	8.98 ± 0.10^A,c^	2.12 ± 0.24^A,c^
CG‐NEN	1	49.61 ± 1.09^A,a^	12.72 ± 0.10^A,a^	8.46 ± 0.23^A,a^	—
	4	49.43 ± 0.88^A,a^	12.59 ± 0.16^A,B,a^	8.50 ± 0.19^A,a,b^	0.23 ± 0.23^B,b^
	8	49.01 ± 1.23^A,a^	12.24 ± 0.22^b,c,a^	8.69 ± 0.11^A,c^	0.80 ± 0.35^B,c,d^
	12	48.32 ± 1.10^A,a^	11.96 ± 0.21^C,a^	8.93 ± 0.27^A,c^	1.59 ± 0.41^A,c^

*Note:* Values represent mean (*n* = 3) ± SD. Small and big different letters indicate significant difference among samples and storage period at 5% level of probability, respectively.

#### 3.3.4 Microbial Load

The microbial load of different coated burger treatments is given in Table 7. At the beginning of the storage period, VMBC and PBC of different burger treatments were in the range of 3.95–4.02 log CFU/g and 3.17–3.25 log CFU/g, respectively, and coating the burgers with CG‐based solutions on this day did not show a significant effect on the number of these bacteria. On this day, the counts of LAB, coliforms, molds, and yeasts of the control sample were 2.71, 1.37, and 1.52 log CFU/g, respectively, and coating the burgers caused a significant decrease in the count of these microorganisms in coated burgers compared to the control. Over time, due to the growth and proliferation of microorganisms in different burger treatments, their number increased significantly (*p* < 0.05), but due to the antimicrobial activity of the coatings used in this study, the intensity of growth and proliferation of this microorganism in coated burgers, especially with solutions enriched with SE and NE, decreased significantly compared to the control. At the end of the storage period, the highest counts of VMBC, PBC, LAB, coliforms, and molds and yeast were in the control sample (8.73, 8.19, 7.18, 4.41, and 4.90 log CFU/g, respectively), and the burger coated with CG solution without essential oil had the next highest (6.92, 6.73, 6.05, 3.13, and 3.68 log CFU/g, respectively). Treatments coated with SE and NE‐enriched solutions had significantly lower microbial load on the last day (*p* < 0.05), and the lowest number of bacteria was observed on this day in burgers coated with SE nanoemulsion solution (5.34, 4.69, 4.33, and 1.89 log CFU/g). In terms of mold and yeast counts, the lowest number on the last day was observed in burgers coated with NE nanoemulsion solution (2.54 meq/kg).

**TABLE 7:** Microbial load count (log CFU/g) of beef burgers during the storage period.

Samples	Storage time (Day)	VMBC	PBC	LAB	Coliforms	Molds and yeasts
Control	1	4.02 ± 0.05^D,a^	3.25 ± 0.03^D,a^	2.71 ± 0.01^D,a^	1.37 ± 0.04^D,a^	1.52 ± 0.02^D,a^
	4	5.93 ± 0.02^C,a^	4.81 ± 0.04^C,a^	3.69 ± 0.03^C,a^	1.69 ± 0.02^C,a^	2.39 ± 0.04^C,a^
	8	7.48 ± 0.05^B,a^	6.80 ± 0.04^B,a^	5.90 ± 0.05^B,a^	2.95 ± 0.02^B,a^	3.56 ± 0.02^B,a^
	12	8.73 ± 0.04^A,a^	8.19 ± 0.05^A,a^	7.18 ± 0.01^A,a^	4.41 ± 0.03^A,a^	4.90 ± 0.03^A,a^
CG	1	3.95 ± 0.03^D,b^	3.23 ± 0.03^D,a^	2.59 ± 0.03^D,b^	1.25 ± 0.03^D,b^	1.45 ± 0.01^D,b^
	4	4.37 ± 0.01^C,b^	4.01 ± 0.01^C,b^	3.10 ± 0.04^C,b^	1.42 ± 0.01^C,b^	1.83 ± 0.03^C,b^
	8	5.61 ± 0.05^B,b^	5.46 ± 0.05^B,b^	4.82 ± 0.03^B,b^	2.04 ± 0.02^B,b^	2.49 ± 0.05^B,b^
	12	6.92 ± 0.01^A,b^	6.73 ± 0.01^A,b^	6.05 ± 0.05^A,b^	3.13 ± 0.01^A,b^	3.68 ± 0.01^A,b^
CG‐SE	1	3.99 ± 0.03^D,a^	3.19 ± 0.05^D,a^	2.56 ± 0.03^C,b^	1.24 ± 0.03^C,b^	1.38 ± 0.03^C,c^
	4	4.13 ± 0.05^C,c^	3.56 ± 0.02^C,d^	2.85 ± 0.01^C,c^	1.27 ± 0.04^C,c^	1.48 ± 0.07^C,c,d^
	8	4.86 ± 0.02^B,d^	3.97 ± 0.02^B,e^	3.35 ± 0.01^B,d^	1.56 ± 0.03^B,d^	1.86 ± 0.02^B,c^
	12	5.51 ± 0.01^A,e^	5.22 ± 0.03^A,d^	4.50 ± 0.04^A,d^	2.14 ± 0.02^A,d^	2.70 ± 0.04^A,c^
CG‐SEN	1	3.95 ± 0.04^D,a^	3.17 ± 0.06^D,a^	2.53 ± 0.03^D,b^	1.20 ± 0.03^C,b^	1.40 ± 0.05^C,b,c^
	4	4.15 ± 0.02^C,c^	3.48 ± 0.02^C,e^	2.81 ± 0.03^C,c,d^	1.24 ± 0.01^C,c^	1.47 ± 0.02^C,c^
	8	4.79 ± 0.04^B,e^	3.80 ± 0.04^B,f^	3.27 ± 0.02^B,e^	1.50 ± 0.01^B,e^	1.83 ± 0.03^B,c^
	12	5.34 ± 0.03^A,f^	4.69 ± 0.03^A,f^	4.33 ± 0.05^A,e^	1.89 ± 0.04^A,f^	2.62 ± 0.02^A,d^
CG‐NE	1	3.97 ± 0.01^D,a^	3.21 ± 0.03^D,a^	2.56 ± 0.02^D,b^	1.25 ± 0.02^C,b^	1.38 ± 0.03^C,c^
	4	4.18 ± 0.02^C,c^	3.79 ± 0.03^C,c^	2.85 ± 0.01^C,c^	1.26 ± 0.02^C,c^	1.42 ± 0.02^C,d,e^
	8	4.93 ± 0.01^B,c^	4.52 ± 0.02^B,c^	3.46 ± 0.03^B,c^	1.63 ± 0.03^B,c^	1.77 ± 0.01^B,d^
	12	5.90 ± 0.04^A,c^	5.68 ± 0.05^A,c^	4.71 ± 0.02^A,c^	2.27 ± 0.04^A,c^	2.65 ± 0.02^A,c,d^
CG‐NEN	1	3.98 ± 0.03^D,a^	3.17 ± 0.06^D,a^	2.56 ± 0.02^D,b^	1.23 ± 0.03^C,b^	1.33 ± 0.03^C,c^
	4	4.15 ± 0.02^C,c^	3.53 ± 0.05^C,d,e^	2.78 ± 0.02^C,d^	1.26 ± 0.02^C,c^	1.38 ± 0.02^C,e^
	8	4.85 ± 0.02^B,d,e^	4.04 ± 0.02^B,d^	3.39 ± 0.04^B,c,d^	1.61 ± 0.02^B,c,d^	1.69 ± 0.01^B,e^
	12	5.67 ± 0.01^A,d^	5.10 ± 0.04^A,e^	4.49 ± 0.02^A,d^	1.96 ± 0.02^A,e^	2.54 ± 0.01^A,e^

*Note:* Values represent mean (*n* = 3) ± SD. Small and big different letters indicate significant difference among samples and storage period at 5% level of probability, respectively.

Abbreviations: LAB, lactic acid bacteria; PBC, psychrophilic bacteria count; VMBC, viable mesophilic bacteria count.

During the storage period of meat and meat products, microbial load analysis is essential because these products are favorable for the growth of microorganisms. As a result of the growth of microorganisms, enzymes are secreted that can intensify the oxidation of proteins and lipids, causing spoilage of these products and the development of discoloration, odor, and off‐flavor in them (Hu et al. 2024). Chitosan has intrinsic antimicrobial activity, and this activity is due to its polycationic nature, which leads to the destruction of cell membranes and the leakage of intracellular contents and inhibits mRNA synthesis (Díaz‐Montes and Castro‐Muñoz 2021). Mahalleh et al. (2020) also showed that free and encapsulated extracts of *N. binaludensis* controlled the growth of mold and yeast in yogurt and reported its effect to be higher than that of the chemical preservative potassium sorbate. Fathimoghaddam et al. (2020) observed strong antimicrobial activity of SE and stated that this activity was concentration dependent. One of the main advantages of the encapsulation process is the gradual and controlled release of bioactive compounds during storage, thereby maintaining the antimicrobial bioactive additive performance during the product storage period (Jafarinia et al. 2022). In addition, encapsulated essential oils are smaller in size compared to the free form of the essential oil and therefore have a higher surface to volume ratio, and therefore their interaction with the bacterial cell wall and membrane is greater and therefore they can also exhibit stronger antimicrobial activity (Lim et al. 2023). Ashrafi et al. (2024) also significantly reduced the microbial load of frozen burgers compared to the control using an active biopolymer coating containing a nanoemulsion of *Rosa canina* L. extract. These researchers also found that antimicrobial activity was significantly improved by increasing the level of nanoemulsion in the coating solutions. Reduction of microbial load of beef during cold storage using chitosan coating containing lemon essential oil was also reported in a study by Isvand et al. (2024). These results were consistent with the findings of Farokhzad et al. (2023) regarding the higher antimicrobial effect of coating containing encapsulated rosemary essential oil compared to the free form of the essential oil. Comparable findings were reported by Yaghoubi et al. (2023), who showed that the incorporation of essential oil together with natural nanoparticles significantly improved microbial stability and preserved the quality attributes of frankfurter‐type sausages during refrigerated storage. Similarly, Safari et al. (2023) demonstrated that double gelatin/chitosan nanoparticle‐calcium alginate coatings effectively suppressed microbial proliferation and improved the storage stability of chicken breast, confirming the potential of biopolymer‐based active coating systems for refrigerated meat preservation.

#### 3.3.5 Sensory Evaluation

**FIGURE 5:**
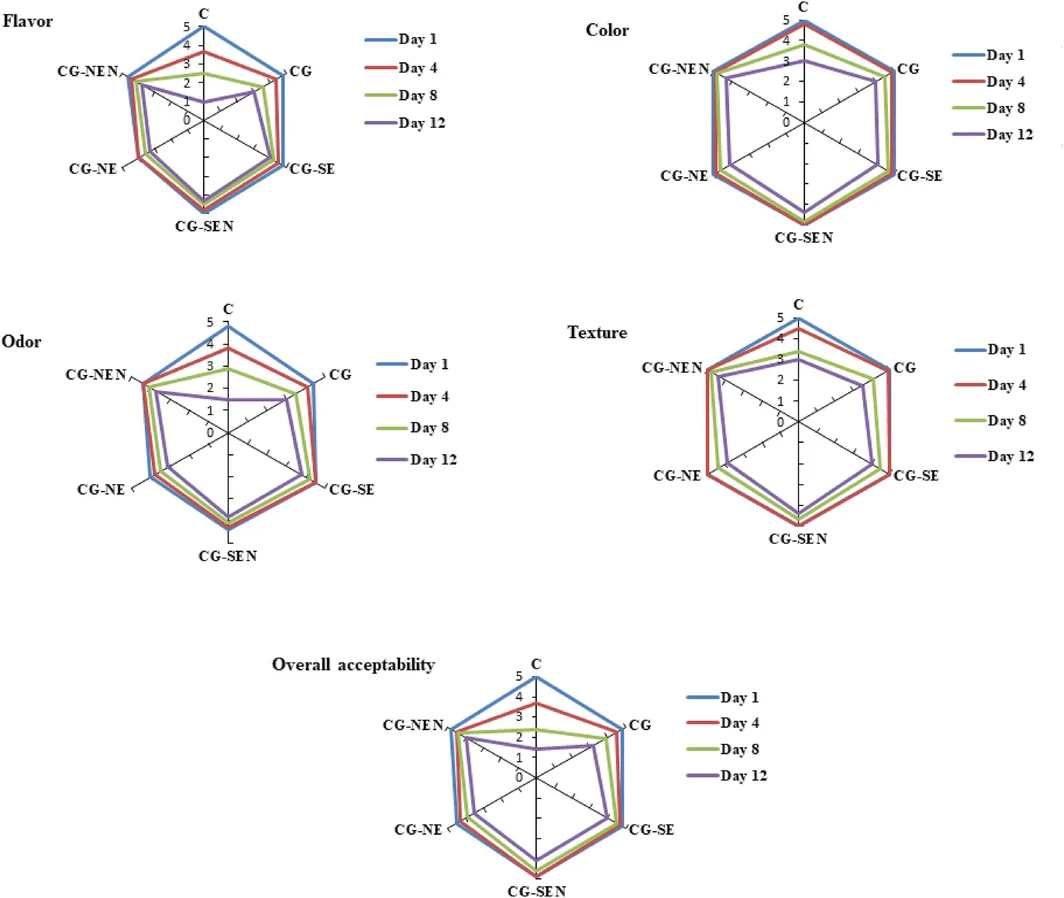
Comparison of sensory characteristics scores of beef burgers during the storage period.

The changes in the mean sensory scores of coated burgers during the 12‐day storage period under refrigerated conditions are shown in Figure 5. At the beginning of the storage period, the control sample obtained a full taste score (5.00), and there was no statistically significant difference between its taste score and the scores of burgers coated with CG solution without essential oil and solutions containing free and microencapsulated savory essential oil. The use of coatings containing NE, especially its free form, significantly reduced the taste score of burgers compared to the control (*p* < 0.05). All burger treatments obtained a full texture and color score on the first day (5.00), and their smell score was in the good range (above 4). During the storage period, the flavor and odor scores of different burger treatments gradually decreased, but the greatest decrease in flavor and odor scores was related to the control sample and burger coated with CG solution without essential oil, respectively. The decrease in the flavor and odor scores of burgers over time can be related to the oxidation of lipids and the production of secondary oxidation products (such as aldehydes, alcohols, esters, etc.) as well as the activity of microorganisms and the production of their by‐products. The decrease in the color score of burgers over time was also observed due to the oxidation of meat pigments and the production of brown met myoglobin. The decrease in moisture content and the activity of microorganisms can also cause changes in the color of meat products. Over time, the texture score of the control sample decreased significantly from the first to the last day, but a decrease in the texture score of burgers coated with CG solutions was observed from the eighth day. The decrease in the texture score of burgers over time is related to the gradual loss of moisture content and also the degradation of proteins by protease enzymes secreted by the activity of microorganisms. Due to the antimicrobial and antioxidant activities of the coatings used in this study, as well as their effect on moisture retention, the severity of the decline in the scores of various sensory properties and overall acceptance of the coated burgers during storage was significantly reduced compared to the control. At the end of the storage period, the lowest scores of sensory properties and sensory acceptance were for the control sample, and among the coated burgers, the burger coated with the CG solution without essential oil obtained the lowest sensory scores on the last day. In general, the burgers coated with solutions containing free essential oil and SE nanoemulsion, as well as the burger coated with a solution containing NE nanoemulsion, obtained the highest overall acceptance score, and their scores were higher than 4. In line with the results of this study, the effect of free and encapsulated extract of *N. binaludensis* on maintaining the sensory acceptance of dough was also reported by Mahalleh et al. (2020). Isvand et al. (2024) also showed in agreement that chitosan coating containing lemon essential oil significantly preserved the sensory properties of beef during storage under refrigerated conditions. Gaba et al. (2025) also reported that chitosan‐based active coatings containing thyme and oregano essential oils were able to significantly reduce the severity of degradation in the sensory properties of beef burgers during storage under refrigerated conditions compared to the control. Peighambardoust et al. (2022) also demonstrated that active chitosan coatings incorporating yarrow essential oil effectively delayed quality deterioration and extended the shelf life of chicken fillets, further highlighting the practical applicability of active biopolymer‐based preservation systems for perishable meat products.

## 4 Conclusion

This study confirms that *Satureja* and *Nepeta* essential oils are rich sources of bioactive compounds, with SE demonstrating superior TPC and stronger antioxidant and antimicrobial properties than NE. While nanoencapsulation reduced the TPC of both oils, it significantly enhanced their antimicrobial efficacy, likely due to increased surface area. The incorporation of these essential oils into chitosan‐gelatin (CG) coatings markedly improved the coatings' antioxidant and antimicrobial performance. Coated burgers exhibited significantly reduced microbial growth, with total viable count reductions of up to 2.8 log CFU/g compared to the control on day 12, and maintained their physicochemical properties, effectively delaying spoilage and extending refrigerated shelf life. Notably, the nanoencapsulation process was crucial for preserving the essential oils' functionality during storage, as the nanoemulsified forms exhibited higher antimicrobial activity than the free oils on the final storage day. Furthermore, the CG coatings, particularly those containing the Satureja essential oil nanoemulsion (CG‐SE‐NE), effectively preserved the sensory acceptability of the burgers throughout refrigerated storage. The coating containing CG‐SE‐NE showed the greatest overall effectiveness in maintaining product quality, microbial safety, and sensory attributes. This research demonstrates that CG‐based coatings incorporating encapsulated essential oils are an effective preservation strategy for refrigerated beef burgers. Although the coating system may have potential for application to other high‐moisture muscle foods that undergo similar microbial and oxidative deterioration, such applicability remains a hypothesis and should be verified experimentally for each food matrix because differences in composition, surface characteristics, and storage conditions may influence coating performance. Future studies should focus on optimizing coating composition (e.g., polymer ratio, essential oil concentration, and nanoemulsion characteristics), validating the antimicrobial and antioxidant mechanisms through controlled release and migration analyses, and evaluating coating performance in different food matrices under commercial processing and storage conditions. Such investigations would provide the mechanistic and application‐specific evidence required for broader industrial implementation.

## Author Contributions

**Parnian Pezeshki:** investigation, validation, data curation. **Mahsa Nakhaei:** investigation, writing – original draft, formal analysis. **Homa Baghaei:** conceptualization, validation, supervision, methodology. **Ahmadreza Abedinia:** investigation, writing – review and editing, visualization, software, data curation. **Fariborz Nahidi:** investigation, validation, data curation.

## Ethics Statement

As no human ethics committee or formal documentation process was available, we declare that appropriate protocols were used to protect the rights and privacy of all participants during the conduct of the study, including no coercion to participate, full disclosure of study requirements and risks, written or verbal consent of participants, no release of participant data without their knowledge, and the ability to withdraw from the study at any time.

## Consent

The authors have nothing to report.

## Conflicts of Interest

The authors declare no conflicts of interest.

## Data Availability

The data that support the findings of this study are available from the corresponding author upon reasonable request.
